# High-Performance Flexible Hybrid Silica Membranes with an Ultrasonic Atomization-Assisted Spray-Coated Active Layer on Polymer for Isopropanol Dehydration

**DOI:** 10.3390/membranes14070154

**Published:** 2024-07-12

**Authors:** Mingjia Liao, He Guan, Hongfen Zuo, Guannan Ren, Genghao Gong

**Affiliations:** 1 Department of Chemical Engineering, Chongqing Chemical Industry Vocational College, Chongqing 401228, China; 2 State Key Laboratory of Separation Membranes and Membrane Processes, School of Materials Science and Engineering, Tiangong University, Tianjin 300387, China; 3 College of Textile and Garment, Yantai Nanshan University, Yantai 265713, China; 4 Cangzhou Institute of Tiangong University, Cangzhou 061000, China

**Keywords:** hybrid silica membrane, ultrasonic spraying, pervaporation, polyimide substrate

## Abstract

Organic–inorganic hybrid silica materials, incorporating an organic group bridging two silicon atoms, have demonstrated great potential in creating membranes with excellent permselectivity. Yet, the large-scale production of polymer-supported flexible hybrid silica membranes has remained a significant challenge. In this study, we present an easy and scalable approach for fabricating these membranes. By employing a sol–gel ultrasonic spray process with a single-pass method, we deposited a thin and uniform hybrid active layer onto a porous polymer substrate. We first optimized the deposition conditions, including substrate temperature, the binary solvent ratio of the silica sol, and various ultrasonic spray parameters. The resulting flexible hybrid silica membranes exhibited exceptional dehydration performance for isopropanol (IPA)/water solutions (IPA: 90 wt%) in the pervaporation process, achieving a water flux of 0.6 kg/(m^2^ h) and a separation factor of around 1300. This work demonstrates that the single-pass ultrasonic spray method is an effective strategy for the large-scale production of polymer-supported flexible hybrid silica membranes.

## 1. Introduction

Conventional separation methods like distillation and evaporation typically consume vast amounts of energy, resulting in significant fossil fuel usage and severe environmental impacts [1,2]. In contrast, membrane-based separation technologies provide high separation efficiency, low energy consumption, compact design, and environmental friendliness, making them highly attractive for molecular separation in the chemical industry. Specifically, for energy-intensive separation processes, membrane-based methods can reduce energy consumption by up to 90% compared to traditional distillation, which accounts for 10–15% of global energy use [3]. Pervaporation (PV) and vapor permeation (VP) are membrane-based processes commonly used for separating binary or multicomponent organic mixtures, especially azeotropes and mixtures with close boiling points [4]. For the dehydration of ethanol and isopropanol, PV and VP systems are particularly favored due to their superior separation performance and cost-efficiency. The success of these systems relies on high-quality membranes with excellent thermal and chemical stability and optimal permselectivity [5,6].

In recent decades, the quest for high-performance membranes for solvent dehydration has led to the extensive exploration of various microporous materials, including polymers, inorganic substances, and organic–inorganic hybrid materials [4,5,6]. Among them, a new class of organic–inorganic hybrid silica membranes containing a bridging organic group between two silicon atoms (≡Si-R-Si≡, R = bridging organic group) have garnered significant attention for separation applications due to their exceptional molecular sieving capabilities, customizable pore sizes, and remarkable thermal and chemical resistance [7,8]. These desirable properties are sourced from the homogeneous incorporation of diverse organic bridges via covalent bonding in the silica networks. For example, researchers have successfully prepared hybrid silica membranes using 1,2-bis(triethoxysilyl)ethane (BTESE) through a sol–gel process [9]. These BTESE-derived silica networks, which feature an ethylene bridging group (-CH_2_-CH_2_-) between two silicon atoms, have demonstrated impressive hydrothermal stability, maintaining their performance for over 1000 days during the continuous pervaporative dehydration of n-butanol at 150 °C [10]. Additionally, BTESE membranes fabricated using a “spacer” technique have shown enhanced hydrothermal stability and high hydrogen permeability in gas separations [11]. Our team was the first to apply BTESE membranes to the reverse osmosis (RO) desalination of NaCl solutions, showcasing superior chlorine tolerance across a wide range of chlorine concentrations (35,000 ppm·h) and stability in both inorganic (nitric) and organic (acetic) acids [12]. Despite these advancements, the field of hybrid silica membrane fabrication remains dominated by ceramic membranes. These membranes are typically prepared on flat or tubular ceramic supports, but their widespread application is hindered by poor reproducibility, the complexity of inorganic membrane preparation, and the high cost of ceramic supports [7,8,9,10,11,12,13,14,15].

A new trend in separation membrane technology involves creating layered hybrid membranes with a thin, dense hybrid silica film on a porous, flexible polymer support. These membranes offer excellent reproducibility, lower costs, and impressive separation performance compared to ceramic membranes. For example, Ngamou et al. used expanding thermal plasma chemical vapor deposition (ETP-CVD) to deposit a BTESE-derived silica layer onto porous polyamide-imide substrates [16]. The resulting hybrid silica membrane performed as well as ceramic membranes for the pervaporative dehydration of an n-butanol–water mixture. However, the ethane bridges (CH_2_-CH_2_) in the silica networks, essential for hydrothermal stability, were only partially retained (about 30%) due to decomposition by reactive oxygen species during the ETP-CVD process. Subsequently, several methods such as sol–gel spin coating [17], flow-induced deposition [18], and air spray coating [19] have been reported for the high-quality deposition of hybrid silica separation layers on porous polymer supports. However, the aforementioned methods are either unsuitable for large-scale membrane production or have issues with reproducibility. Although air spray coating has been used for the large-area deposition of hybrid silica separation layers, the high-pressure airflow can disturb the liquid film, resulting in the poor uniformity of the formed hybrid silica separation layer.

In this work, we proposed an ultrasonic atomization-assisted spraying (ultrasonic spraying) approach for the preparation of flexible hybrid silica membranes. A thin and uniform hybrid silica active layer was deposited onto a porous polyimide UF supporting membrane. The ultra-low carrier gas pressure prevents disturbances to the coating surface caused by airflow. Simultaneously, ultrasonic treatment produces spray micro-droplets of relatively uniform size, which helps form a consistent liquid film layer, facilitating the uniform and defect-free deposition of hybrid silica separation layers. Moreover, the ultrasonic spray conditions were further investigated to achieve the high-quality deposition of a thin, uniform, and defect-free hybrid silica active layer on polymer substrates. The PV dehydration of an IPA/H_2_O solution was carried out to assess the separation performance of the polymer-supported flexible hybrid silica membrane and to further optimize membrane fabrication.

## 2. Experimental Section

### 2.1. Materials

The bridged silsesquioxane precursor bis(triethoxysilyl)ethane (BTESE) was obtained from Fluorochem Co., Ltd. (Glossop, UK). Lenzing P84 polyimide (Granlat/SG, Mw = 100,000 g mol^−1^) was purchased from Ensinger Sintimid GmbH (Linz, Austria). Dimethylformamide (DMF) and other chemicals including polyethylene glycol 400 (PEG400, Mw = 327 g mol^−1^), ethanol, isopropanol, and n-propanol were purchased from Tianjin Kermel Chemical Reagent Co., Ltd. (Tianjin, China). All chemicals were utilized without further purification.

### 2.2. Synthesis of BTESE Polymeric Sols

A polymeric sol of 1,2-bis(triethoxysilyl)ethane (BTESE) was synthesized through hydrolysis and condensation [19] in a concoction of water, HCl, and isopropanol (IPA). Briefly, a specific amount of BTESE and deionized (DI) water was added to IPA. Upon the addition of a certain amount of HCl into this solution, the mixture was stirred for 1.5 h in a sealed glass bottle at 60 °C. Finally, we obtained a 10 wt% BTESE sol, which is approximately equal to 0.7 mol% BTESE (the molar ratio of BTESE:H_2_O:HCl = 1:60:0.1), which then was diluted with IPA/H_2_O to achieve levels of 3.0, 4.0, and 5.0 wt% of BTESE. The resulting BTESE sol was then stored at 4 °C until further use. The specific hydrolysis (Equation (1)) and condensation (Equation (2)) reactions of the BTESE sol are as follows:

(EtO)_3_Si–C_2_H_4_–Si(OEt)_3_ + H_2_O ↔ (EtO)_3_Si–C_2_H_4_–Si–OH + EtOH (1)

(EtO)_3_Si–C_2_H_4_–SiOH + HOSi–C_2_H_4_–Si(EtO)_3_ ↔ (EtO)_3_Si–C_2_H_4_–Si–O-Si–C_2_H_4_–Si(EtO)_3_ + H_2_O (2)

### 2.3. Preparation of Polyimide Ultrafiltration Membrane

**Figure 1:**
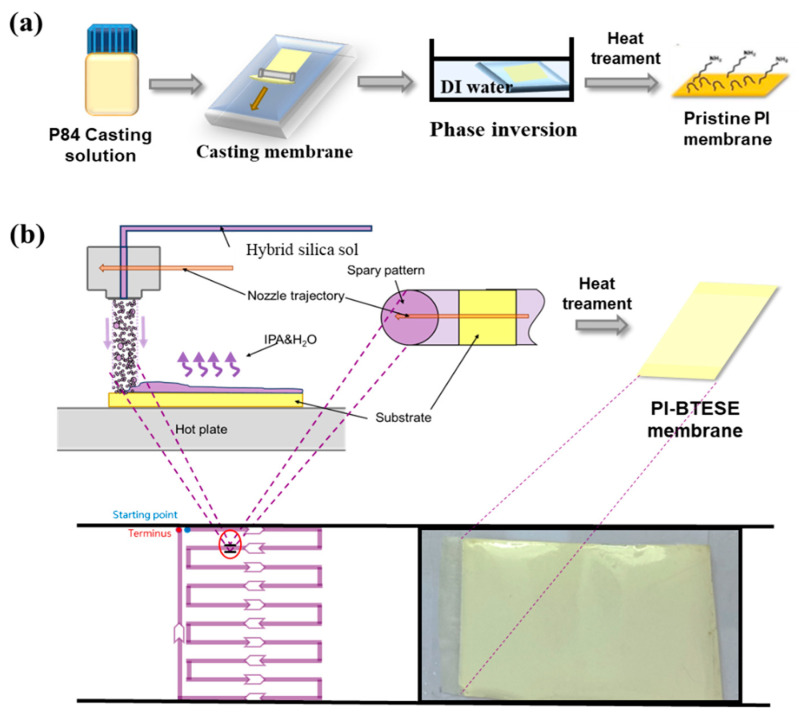
Schematic illustration for the preparation of (**a**) the PI UF membrane using the phase inversion method and (**b**) the flexible hybrid silica membrane with the BTESE layer as the selective layer via ultrasonic spraying followed by heat treatment.

The polyimide (PI) ultrafiltration (UF) support was prepared following a previously reported method [20]. Briefly, as shown in Figure 1a, a 22 wt% P84 solution in dimethylformamide (DMF) was cast onto non-woven polyester fabric using a casting knife with a 250 μm gap. The cast solution was immediately immersed in a deionized (DI) water bath for 15 min to form the PI UF supporting membrane. The membrane was then thoroughly washed with DI water to remove any residual DMF. To ensure that most of the large pores were filled, the bottom of the PI UF support was conditioned with a solution of PEG400 (pore-retaining agent) in isopropanol (3:2 volume ratio of PEG400 to IPA). After drying at room temperature, the PI UF support was ready to use as the substrate for the composite membrane fabrication.

### 2.4. Fabrication of Flexible Hybrid Silica Membranes

Flexible hybrid silica composite membranes with a BTESE active layer on PI support were fabricated using the sol–gel ultrasonic spraying approach, as depicted in Figure 1b. An A4-sized piece of PI membrane was secured onto the horizontal plate of an ultrasonic spraying system (UAM4000L, Hangzhou Chifei Ultrasonic Equipment Co., Ltd., Hangzhou, China). The BTESE sol was deposited onto the PI support surface using a single-pass method, achieving large-area depositions through a zigzag nozzle trajectory. The nozzle–substrate distance was set to 6 cm, and the nozzle velocity to 10,000 mm/min. Following a similar approach to our previous report [19], the effective deposition area for the single-pass spray coating matched the step width of the zigzag trajectory to ensure uniform and complete coverage. After drying, the resulting PI-supported BTESE-derived silica composite (BTESE/PI) membranes were heat-treated at 150 °C for 10 min. Finally, the BTESE/PI membrane was washed with DI water to remove the pore-retaining agent.

### 2.5. Characterization

The membrane sample surface was measured using attenuated total reflectance Fourier transform infrared spectroscopy (ATR-FTIR, Bruker, TENSOR II, Karlsruhe, Germany). The chemical composition of membrane surfaces was detected using a K-Alpha X-ray Photoelectron Spectrometer System (XPS, Thermo Fisher, K-alpha, Waltham, MA, USA). Thermogravimetric (TG) analysis (DTG-60, Shimadzu Co., Tokyo, Japan) was applied to the PI after pretreatment at 100 °C for 2 h under an air flow of 30 cc min^−1^. The temperature was increased at a ramping rate of 10 °C min^−1^ to 800 °C. The surface and cross-sectional morphologies of the membranes were imaged using a field-emission scanning electron microscope (SEM, Zeiss, Gemini 500, Jena, Germany) with the help of Energy-Dispersive X-ray spectroscopy (EDX) qualitative analysis. The topographies of the BTESE active layer were characterized using an atomic force microscope (AFM, Bruker, Icon, Germany). 

### 2.6. Pervaporation Dehydration Experiments

**Figure 2:**
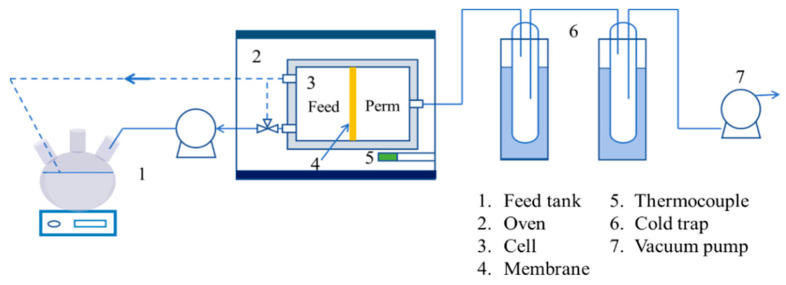
Schematic diagrams of the PV experimental setup.

Pervaporation (PV) dehydration experiments were conducted using the setup illustrated in Figure 2, with an effective membrane surface area of approximately 2.27 cm^2^. The feed solution, containing 10% water in IPA, was circulated through a coil within the membrane cell housed in an oven. The retentate stream was returned to the feed tank open to the atmosphere, while the permeate stream was maintained at less than 1.0 kPa using a vacuum pump. Species concentrations in the feed and permeate were measured using a gas chromatograph (GC-7890B, Agilent Technology, Santa Clara, CA, USA) equipped with a thermal conductivity detector.

The PV dehydration performance of membranes was evaluated in terms of the permeation flux (*J*(i): kg m^−2^h^−1^) of the i component and separation factor (*α*), calculated as follows:

(3) $$J\left(i\right)=\frac{Q\left(i\right)}{At}$$

where *Q*(i) is the mass of permeated component *i* collected over time *t*, and *A* is the effective membrane area for permeation. The separation factor (α) was calculated using the following equation:

(4) $$\alpha =\frac{Y_{w}/Y_{i}}{X_{w}/X_{i}}$$

where *Y* and *X* are the weight fractions of water (*w*) and isopropanol (*i*) in the permeate and feed solutions, respectively.

## 3. Results and Discussion

### 3.1. Thermal Tolerance of an PI Support Membrane

Typically, organic–inorganic hybrid silica membranes require high-temperature sintering to form a dense silica network [21]. However, polymer support membranes generally cannot withstand such high temperatures. Therefore, the heat resistance of the PI support was investigated firstly, which was critical in determining the curing temperature of BTESE layers on PI supports.

**Figure 3:**
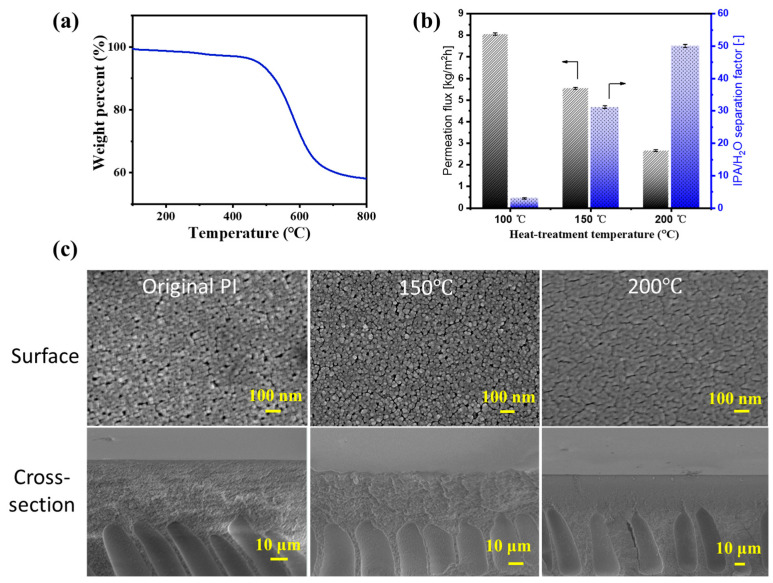
The (**a**) TG curve of PI; (**b**) PV performance of the PI UF membranes as a function of heat-treatment temperature for IPA/water solution (IPA: 90 wt%) at 60 °C; (**c**) SEM images of the surface and cross-section of untreated and treated PI membranes at various temperatures for 10 min (150 and 200 °C).

Figure 3a illustrates the weight loss of the PI (XP84) when subjected to temperatures ranging from 25 to 800 °C. The weight loss occurred above 400 °C, primarily due to the decomposition of the organic components, indicating the excellent thermal stability of the PI support. However, investigating PV dehydration performance for IPA/water mixtures of the PI membrane at different heat-treatment temperatures revealed that as the temperature increased, the permeation flux gradually decreased while the separation factor slightly improved (Figure 3b). Figure 3c shows SEM images of the surface and cross-section of untreated and treated PI membranes at various temperatures. With increasing heat-treatment temperatures, the surface pores of the PI membrane become smaller, and the number of finger-like pores decreases, resulting in a denser surface. Fortunately, at 150 °C, the overall pore structure remains largely unaffected, with only moderate surface pore shrinkage. This slight reduction in surface pore size may prevent the excessive leakage of the hybrid silica sol during ultrasonic spraying, thereby promoting the uniform and defect-free deposition of the BTESE layer on its surface. Therefore, unless otherwise specified, the heat-treatment temperature is set to 150 °C in the subsequent experiments.

### 3.2. Ultrasonic Spraying of BTESE Sol on the Porous PI Support

While spray coating is commonly used for large-area deposition, achieving uniformity and defect-free coverage on polymer substrates remains challenging. As shown in Figure 1b, the single-pass spray coating process involves spraying micrometer-sized droplets onto the substrate. These droplets spread and merge to form a continuous liquid film, which solidifies into a thin layer upon solvent evaporation. To ensure uniform and complete coverage, the good wettability of the liquid is crucial. Low-surface-tension droplets coalesce into a continuous film rather than forming individual spherical caps. Additionally, good wettability requires less liquid volume to cover the substrate fully and ensures more uniform evaporation across the surface. In this work, the hybrid silica sol was composed of a binary solvent system with low-surface-tension IPA and high-surface-tension water. This kind of two-solvent system (i.e., when the solvent with low surface tension is characterized by a higher vapor pressure) produces favorable spreading capabilities due to the Marangoni velocity (Marangoni flows are driven by surface tension gradients, which generate an advancing velocity at the droplet edge, allowing it to spread and cover previously unwetted areas). According to Marangoni velocity [22].

(5) $$V_{c}^{2}\left(x\right)=\frac{1}{2\eta \left(x\right)}\frac{d\gamma }{dx}x\left(1-x\right)\left(-A_{l}\alpha_{l}+A_{h}\alpha_{h}\right)$$

where *η* is the viscosity of the liquid film, *γ* represents the surface tension, *x* is the volume fraction of the low-surface-tension solvent, *A_l_* and *A_h_* denote the evaporation velocities, and *α_l_* and *α_h_* are the activity coefficients of the low- and high-surface-tension solvents, respectively. 

**Figure 4:**
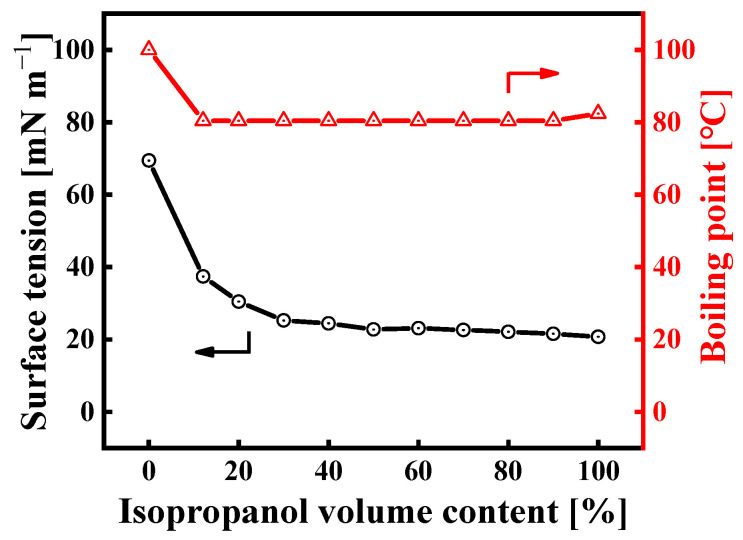
Boiling point and surface tension of IPA/water mixtures at 25 °C as a function of IPA volume content (the boiling point data are from the literature [24]).

It is clear that the main factors affecting Marangoni velocity include surface tension, the volume fraction of the low-surface-tension liquid, and the evaporation rate of the binary solvent. The surface tension and boiling point of IPA/H_2_O mixtures at 25 °C vary with the IPA content, as shown in Figure 4. Adding a small amount of IPA significantly reduces the surface tension of the mixture. When the IPA content is between 50 and 90 vol%, the surface tension of the mixture remains almost stable at 21~23 mN/m, which is promising for achieving a good spreading of the sol on the substrate surface. As a result, this IPA/H_2_O binary solvent system offers a subversive advantage for spray coating. Not only does it lower the contact angle of the mixture, but it also promotes the spreading of the deposited liquid into areas of the substrate not directly hit by the spray. Moreover, the Marangoni effect plays a key role in smoothing the film surface [23].

#### 3.2.1. The Effect of Binary Solvent Ratio (IPA/H_2_O) on the Morphology of Hybrid Silica Layers

**Figure 5:**
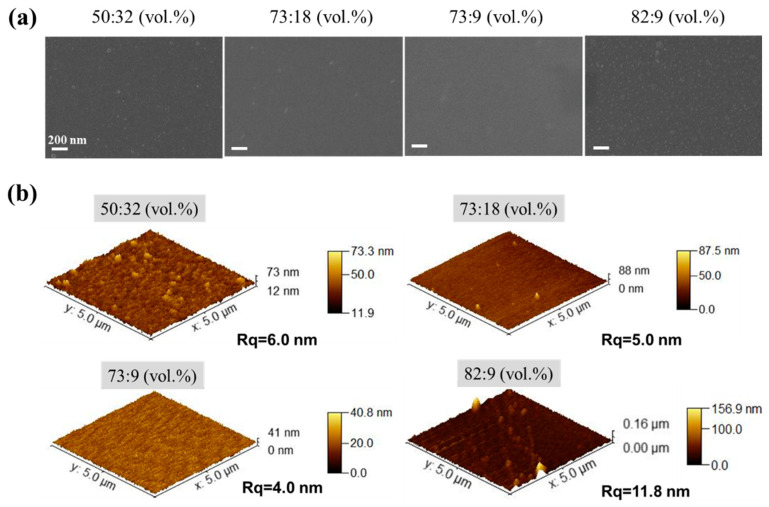
(**a**) SEM and (**b**) AFM images of the surface of BTESE layers deposited on PI support by means of the ultrasonic spraying of BTESE sols (BTESE sol concentration of 5 wt%) prepared with four different IPA/H_2_O volume ratios (vol.%).

Owing to the extremely low viscosity (<5 cp) of the hybrid silica sol [19], achieving a large-area uniform deposition of the hybrid silica functional layer on a porous polymer substrate is highly challenging. Therefore, we first investigated the impact of the binary solvent component ratio of the hybrid silica sol on the BTESE film formation. Figure 5 shows the surface morphology of BTESE layers deposited on PI support by means of the ultrasonic spraying of hybrid silica sol prepared with four different IPA/H_2_O volume ratios (vol.%), observed through SEM (Figure 5a) and AFM (Figure 5b). When the water content is high in the binary solvent system (IPA to water: 55:32 vol.%), there are more agglomerated protrusions on the membrane surface. This is because IPA evaporates faster than water at this ratio, and the diffusion rate of most liquids is lower than the evaporation rate [25]. As a result, the surface tension of the liquid film approaches that of water, causing the liquid film to shrink into microdroplets. These microdroplets dry and form hybrid silica particles on the surface. As the water content in the binary solvent decreased, these raised hybrid silica particles deposited on the surface gradually decreased. When the IPA/H_2_O ratio reached 73:9 vol.%, the hybrid silica particles disappeared from the membrane surface, which became smooth. This is also because, at this ratio, IPA and H_2_O achieve evaporation equilibrium and maintain low surface tension throughout the evaporation process, thereby avoiding the uneven shrinkage of the entire liquid film and ultimately achieving a uniform deposition of the BTESE layer [22].

On the other hand, when the IPA/H_2_O ratio in the sol is 82:9 (vol.%), the membrane surface also exhibits numerous aggregated hybrid silica particles. This is because at this ratio, the surface tension of the sol should be closer to that of pure IPA, and the composition of IPA and water in the sol approaches the azeotropic composition of IPA–water mixture [26]. At this point, the water content in the vapor phase is higher than IPA, causing the adsorption of water vapor on the surface of liquid droplets and generating a local surface tension gradient. This leads to an inward Marangoni effect on the liquid film surface, dispersing the silica sol components into small droplets, resulting in the presence of aggregated hybrid silica particles on the membrane surface [22].

#### 3.2.2. The Effect of the Temperature of the Substrate on the Morphology of Hybrid Silica Layers

**Figure 6:**
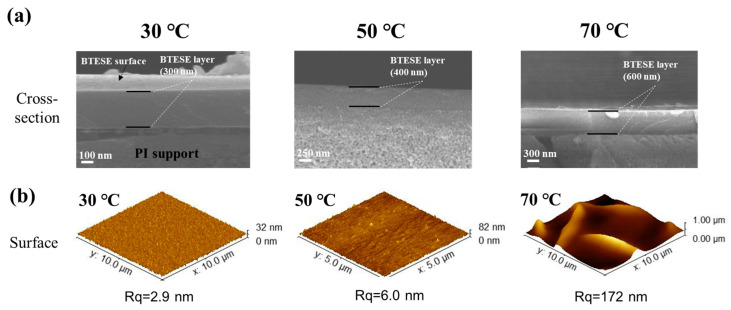
The SEM and AFM images of (**b**) the surface and (**a**) cross-sectional morphologies of BTESE layers at different substrate temperatures of 30, 50, and 70 °C.

The substrate temperature directly influences the rheological properties of the sol micro droplets and the evaporation rate of the solvent in the sol on the PI membrane surface, which is crucial for the BTESE film formation. Figure 6 shows the surface and cross-sectional morphologies of BTESE-derived hybrid silica layer at different substrate temperatures of 30, 50, and 70 °C. The cross-sectional images (Figure 6a) reveal a noticeable increase in the thickness of the BTESE layer as the substrate temperature rises. This is due to changes in the flow and evaporation rates of the sol on the PI substrate surface with varying temperatures. At 70 °C, the solvent in the droplets evaporates rapidly, preventing the droplets from spreading out, resulting in a thicker and more uneven BTESE layer (Figure 6b). When the temperature is lowered to 50 °C, the solvent evaporation rate slows down, allowing more time for the droplets to spread, thus forming a thinner BTESE layer (400 nm thick). At 30 °C, the evaporation and flow rates of the solvent in the sol droplets reach a balance, creating a defect-free and smooth 300 nm thick functional layer on the PI membrane surface. Therefore, the optimal deposition of BTESE layer is achieved at a substrate temperature of 30 °C.

#### 3.2.3. Surface Chemical Properties of PI-Supported Hybrid Silica Composite Membranes

**Figure 7:**
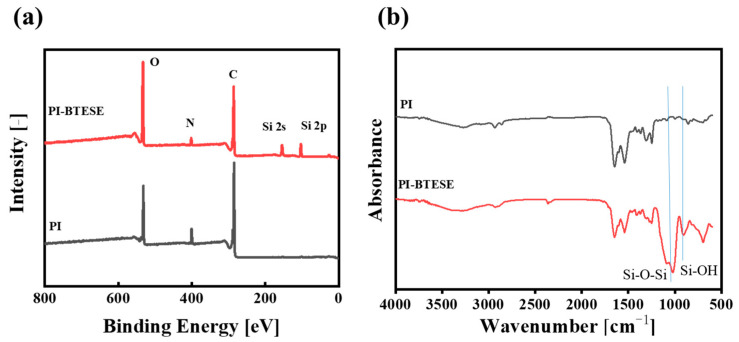
XPS (**a**) and ATR-FTIR (**b**) spectra of the PI and BTESE/PI membranes (sol concentration: 5.0 wt% BTESE with an IPA/H_2_O ratio of 73:9 vol.% and a substrate temperature of 30 °C).

The elemental compositions and chemical structures of a BTESE/PI membrane surface were scrutinized using XPS and ATR-FTIR, respectively, as shown in Figure 7. For the PI membrane, XPS spectra revealed characteristic peaks for O, N, and C at 530.5, 400.5, and 285.5 eV, respectively. In contrast, the BTESE/PI membrane displayed new Si2s and Si2p peaks at 151 and 103.5 eV, respectively, confirming the presence of the BTESE layer on the PI surface (Figure 7a). Additionally, Figure 7b illustrates the ATR-FTIR spectra of PI and BTESE/PI membranes. Beyond the typical absorption bands of the PI support, such as C=O (1720~1700 cm^−1^), C-N (1545 cm^−1^), and =C-O-C= (1240 cm^−1^) stretching vibrations [20], two strong bands corresponding to Si-O-Si and Si-OH groups from the BTESE networks were observed at 1050 and 900 cm^−1^ in the infrared spectrum of the BTESE/PI membrane [18], respectively. Thus, both XPS and FTIR spectra validate the successful formation of a BTESE-derived hybrid silica layer on the PI surface.

### 3.3. PV Performance Test of BTESE/PI Composite Membranes

**Figure 8:**
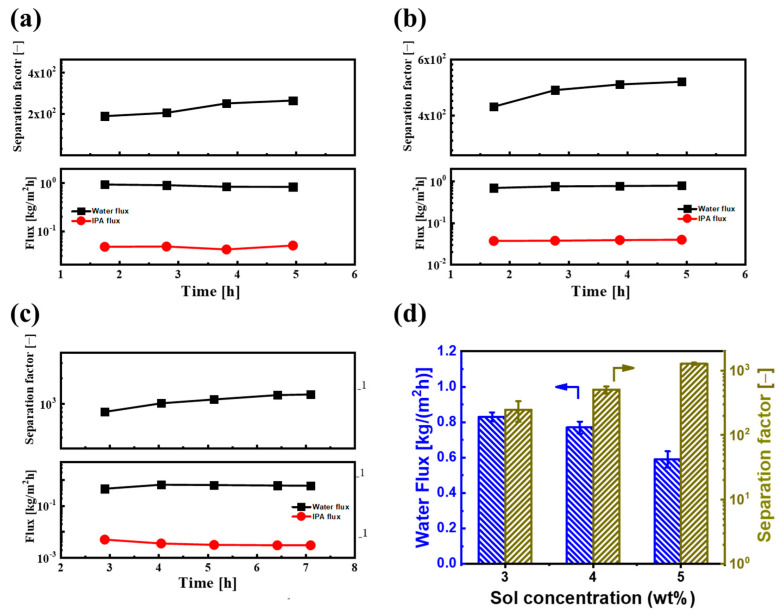
Time courses of PV performances for BTESE/PI membranes prepared with different BETSE concentrations of (**a**) 3, (**b**) 4, and (**c**) 5 wt% for an IPA/water solution (IPA: 90 wt%) at 60 °C; (**d**) the water flux and separation factor of BTESE/PI membranes prepared with different BTESE concentrations ((the PV performances were taken at the last three points of the PV experiment of (**a**–**c**)).

Given that varying sol concentrations can influence the thickness and deposition quality of the BTESE layer on PI supports during the ultrasonic spray process, it is essential to study how sol concentration affects the separation performance of BTESE/PI membranes. Thus, the pervaporation (PV) dehydration performances of BTESE/PI membranes, prepared with different sol concentrations, were assessed using IPA/H_2_O solutions (90 wt% IPA) at 60 °C, as depicted in Figure 8. Figure 8a–c illustrate the PV performance over time for BTESE/PI membranes made with various sol concentrations. After an initial period of moderate fluctuation, the PV performances of all membranes stabilized. Notably, these membranes exhibited relatively stable water flux and a slow decrease in IPA flux during the first 5 h, resulting in a slight increase in the separation factor. This behavior could be attributed to the presence of Si–OH groups within the BTESE structure, stemming from a low degree of condensation reaction in the silanol groups at relatively low curing temperatures (150 °C). It is possible that IPA molecules adsorbed onto or reacted with the Si–OH groups on the pore surfaces of the BTESE layer, leading to a reduction in pore size and thereby hindering IPA molecules from permeating the membrane [27].

**Figure 9:**
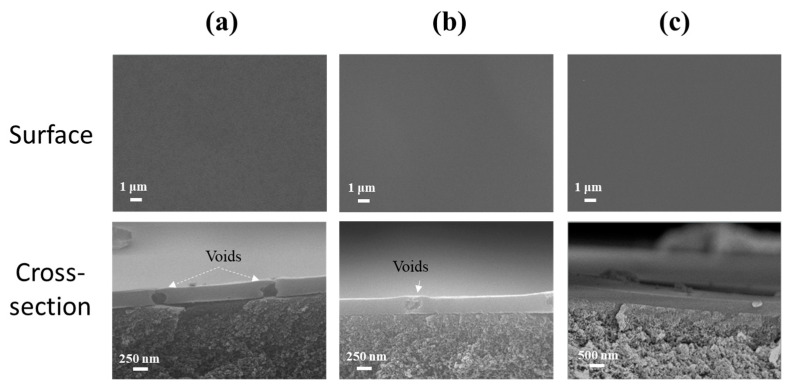
SEM images of surface and cross-sectional morphologies of BTESE/PI membranes prepared with different concentrations of BTESE sols ((**a**) 3.0, (**b**) 4.0, and (**c**) 5.0 wt%).

Figure 8d reveals the water flux and separation factor of membranes prepared with varying BTESE sol concentrations. As the sol concentration increased from 3.0 to 5.0 wt%, the water flux of the BTESE/PI membranes noticeably decreased, while the separation factor steadily rose, reaching a peak of approximately 1280 for the membrane prepared with 5.0 wt% BTESE sol. This suggests that a sol concentration of 5.0 wt% is the minimum threshold for achieving full coverage of the porous PI substrate. Further examination of their cross-sectional structure showed that at lower sol concentrations (3.0 and 4.0 wt%), although no surface defects were visible, some voids were present within the BTESE layer (Figure 9a,b). This may be due to a small amount of sol penetrating into the sublayer of PI supports during the ultrasonic spray process, resulting in a thin BTESE layer with defects. A higher sol concentration led to a thicker BTESE layer, effectively filling the surface porosity of the PI and forming a “composite sublayer” composed of BTESE and PI skin layers (Figure 9c), thereby preventing defect formation in the PI-supported BTESE layer [27] and further enhancing the durability of the BTESE layer on the PI substrate.

**Figure 10:**
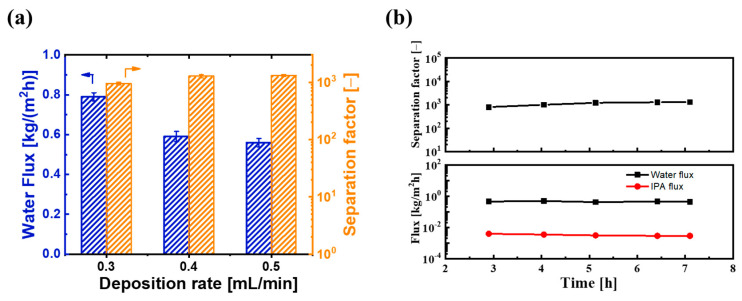
(**a**) PV performances of BTESE/PI membranes prepared with different spray flow rates of the BTESE sol (0.3, 0.4, and 0.5 mL/min), and (**b**) the time course of PV performance for BTESE/PI membranes prepared with spray flow rates of 0.4 mL/min.

On the other hand, we also investigated the effect of ultrasonic spray flow rate on PV performance. Figure 10a shows the water flux and separation factor of BTESE/PI membranes prepared with different spray flow rates. As the spray flow rate of the BTESE sol (5 wt% BTESE) increased from 0.3 to 0.5 mL/min, the water flux decreased from approximately 0.8 to 0.55 kg/(m^2^h), while the separation factor increased from about 800 to around 1305. This could be due to the higher spray flow rate resulting in a thicker BTESE layer with fewer defects. Additionally, compared to the spray flow rate of 0.4 mL/min, the separation factor did not significantly improve at a flow rate of 0.5 mL/min, while the water flux slightly decreased. This indicates that with a sol concentration of 5 wt%, the optimal spray flow rate for the best performance of the BTESE layer is 0.4 mL/min. Under these ultrasonic spray conditions, the BTESE/PI membrane exhibited a stable water permeation flux of approximately 0.6 kg/(m^2^h) and a high separation factor of around 1300 during the 8 h PV test (Figure 10b), thereby indicating its excellent stability. 

Table 1 summarizes the PV separation performance of IPA aqueous solutions using BTESE/PI membranes and other types of PV membranes. Generally, polymeric membranes exhibit low permeation flux and separation factors due to their tendency to swell in many organic solvents. Compared to polymer membranes, BTESE/PI membranes showed a moderate permeation flux and separation factor. Although many inorganic membranes demonstrate both high permeation flux and separation factors, their relatively complicated fabrication processes and high costs still limit their large-scale applications. Therefore, BTESE/PI membranes, which exhibit suitable PV performance and highlight their economic advantages, have significant potential for application. 

## 4. Conclusions

In this work, we unveiled a straightforward and scalable method for fabricating flexible hybrid silica membranes. A thin, uniform, and defect-free hybrid silica active layer was deposited onto a porous PI support using a sol–gel ultrasonic spray technique with a single-pass approach. By meticulously engineering the sol–gel and ultrasonic spray processes, we achieved a high-quality BTESE active layer with optimized thickness and minimal surface roughness on the porous PI support. The resulting flexible hybrid silica membranes, with a BTESE layer thickness of approximately 300 nm, demonstrated exceptional dehydration performance for isopropanol (IPA)–water solutions (IPA: 90 wt%) in the PV process, achieving a water flux of 0.6 kg/(m^2^ h) and a separation factor of 1300. This study highlights the effectiveness of the single-pass ultrasonic spray method for the large-scale fabrication of polymer-supported flexible hybrid silica membranes. 

Although the ultrasonic spray method is suitable for the large-area preparation of hybrid silica separation layers on polymer substrates, it requires high standards for the surface morphology and pore size of the polymer. Therefore, in future work, a thin interfacial layer can be first prepared on the porous polymer substrate, followed by the deposition of the hybrid silica separation layer via ultrasonic spraying. This approach will enhance the versatility of this method on different polymer substrates.

## Figures and Tables

**Table 1:** Summary of PV dehydration performance of IPA/H_2_O solutions for several types of membranes.

Membrane	Water in Feed (wt%)	Temperature (°C)	Flux (g m^−2^ h^−1^)	Separation Factor (α)	Ref.
GO-U-P4	30	25	1479	587.4	[28]
TiO_2_-SiO_2_-ACA	10	50	890	667	[29]
TiO_2_-SiO_2_-ACA	10	50	130	98	
MoS_2_ @PD0.3-TFN	30	25	2870.1	268.6	[30]
PDA/HNTs-PVA-PVAm	20	40	190	479	[31]
BAPP-TMC/PEI	30	25	705	1664	[32]
	30	70	2574	159	
(PEI/GO)14/Cl-TFC	6	50	1336	180	[33]
MOF-303/α-Al_2_O_3_	10	70	250	264	[34]
Chitosan	10	50	178	491	[35]
PVA	10	30	95	77	[36]
BTESE/PI	10	60	600	1300	This work

## Data Availability

Data is contained within the article.
